# Manually curated dataset of catalytic peptides for ester hydrolysis

**DOI:** 10.1016/j.dib.2023.109290

**Published:** 2023-06-05

**Authors:** Patrizia Janković, Erik Otović, Goran Mauša, Daniela Kalafatovic

**Affiliations:** a University of Rijeka, Department of Biotechnology, Rijeka 51000, Croatia; b University of Rijeka, Faculty of Engineering, Rijeka 51000, Croatia; c University of Rijeka, Center for Artificial Intelligence and Cybersecurity, Rijeka 51000, Croatia

**Keywords:** Catalytic peptides, Self-assembly, SMILES, Mechanism of catalysis

## Abstract

Catalytic peptides are low cost biomolecules able to catalyse chemical reactions such as ester hydrolysis. This dataset provides a list of catalytic peptides currently reported in literature. Several parameters were evaluated, including sequence length, composition, net charge, isoelectric point, hydrophobicity, self-assembly propensity and mechanism of catalysis. Along with the analysis of physico-chemical properties, the SMILES representation for each sequence was generated to provide an easy-to-use means of training machine learning models. This offers a unique opportunity for the development and validation of proof-of-concept predictive models. Being a reliable manually curated dataset, it also enables the benchmark for comparison of new models or models trained on automatically gathered peptide-oriented datasets. Moreover, the dataset provides an insight in the currently developed catalytic mechanisms and can be used as the foundation for the development of next-generation peptide-based catalysts.

**Specifications Table**

**Table:** 

Subject	Chemistry
Specific subject area	Catalytic peptides: a compilation of experimentally validated catalytic peptides alongside selected physico-chemical properties and SMILES annotations.
Type of data	Tables in CSV format containing sequences composed of (i) proteinogenic and (ii) non-proteinogenic amino acids as separate lists.
	Figures depicting the distribution of length, composition and properties for catalytic and non-catalytic peptides (for p-NPA hydrolysis)
How the data were acquired	A literature search was conducted to identify all the reported peptide sequences with ester hydrolysis activity, experimentally determined through the para-nitrophenyl acetate (p-NPA) and para-nitrphenyl phosphate (p-NPP) assays.
	Charge, hydrophobicity and isoelectric point were computed with peptides.py library. Sequence similarities were computed in Python programming language and peptide alignment was performed with Needleman-Wunsch alignment algorithm from sckit-bio library. SMILES representation of peptides was generated with RDKit library.
Data format	Raw Analyzed
Description of data collection	Data collection was based on the standard colorimetric assays used for the experimental validation of ester and phosphoester hydrolysis. This reaction was selected to standardize the reported catalytic parameters. Other important features included in the dataset are N- and C- termini modifications, self-assembly propensity and mechanism of action.
Data source location	The data was sourced from publicly available articles [1], [2], [3], [4], [5], [6], [7], [8], [9], [10], [11], [12], [13], [14], [15], [16], [17], [18], [19], [20], [21], [22] found through academic search engines.
Data accessibility	Repository name: Mendeley Data Data identification number: 10.17632/6s9kxj2ndr.2 Direct URL to data: https://data.mendeley.com/datasets/6s9kxj2ndr

## Value of the Data

• The analyzed physico-chemical properties for proteinogenic instances offer insight into existing design strategies important for the development of new catalytic sequences.
• The dataset is based on the catalytic activity towards the same type of reaction (ester hydrolysis) which opens up the opportunity for analysis of sequence-activity relationship and their straightforward comparison.
• The provided SMILES (Simplified molecular-input line-entry system) annotations encode important information about the molecular structure (atoms types, bond, chirality, etc.) which can be useful for machine learning-based predictive modelling.
• The presented manually curated dataset can be used to develop and test proof-of-concept models, but also for the comparison and validation of models trained on automatically gathered peptide datasets.

## 1 Objective

The objective of this dataset is to provide the first comprehensive collection of purely peptidic catalysts including both active and inactive sequences serving as a platform for the design of novel catalytic sequences. Creating comprehensive datasets of peptide catalysts is challenging as their catalytic activities may encompass a wide range of chemical transformations and mechanisms. This dataset focuses on peptide sequences that catalyze ester and phosphoester hydrolysis, two widely-studied and important reactions in biological systems. In order to maintain consistency and comparability among the included peptides, we selected only those that follow Michaelis–Menten kinetics, which provides a reliable framework for determining kinetic parameters such as the catalytic efficiency ($k_{\text{cat}}/K_{\text{M}}$).

## 2 Data Description

We provided a list in CSV format (raw data reposited in Mendeley data), containing 101 positive and negative entries of catalytic peptides active towards the p-NPA and p-NPP substrates [1], [2], [3], [4], [5], [6], [7], [8], [9], [10], [11], [12], [13], [14], [15], [16], [17], [18], [19], [20], [21], [22]. In addition, activities towards other alkyl-based substrates including p-NPB (p-nitrophenyl butyrate), p-NPO (p-nitrophenyl octanoate), p-NPMA (p-nitrophenyl methoxyacetate), p-NPH (p-nitrophenyl hexanoate), p-NPS (p-nitrophenyl salicylate), p-NPProp (p-nitrophenyl propionate), p-NPPalm (p-nitrophenyl palmitate), indoxyl acetate, HPNPP (2-hydroxypropyl-4-nitrophenylphosphate) and BNPP (Bis(4-nitrophenyl)phosphate), were added when available. Peptides were categorized into two tables based on whether they contained proteinogenic or non-proteinogenic amino acids. The tables are comprised of nine columns containing one-letter amino acid codes, N- and C- termini modifications, SMILES (Simplified molecular-input line-entry system) annotations, the substrates tested along with the corresponding catalytic parameter ($k_{\text{cat}}/K_{\text{M}}$), mechanisms of action, ability to form secondary structures and/or self-assemble. The SMILES annotations of peptides without termini modifications are provided for sequences containing only proteinogenic amino acids.

**Fig. 1:**
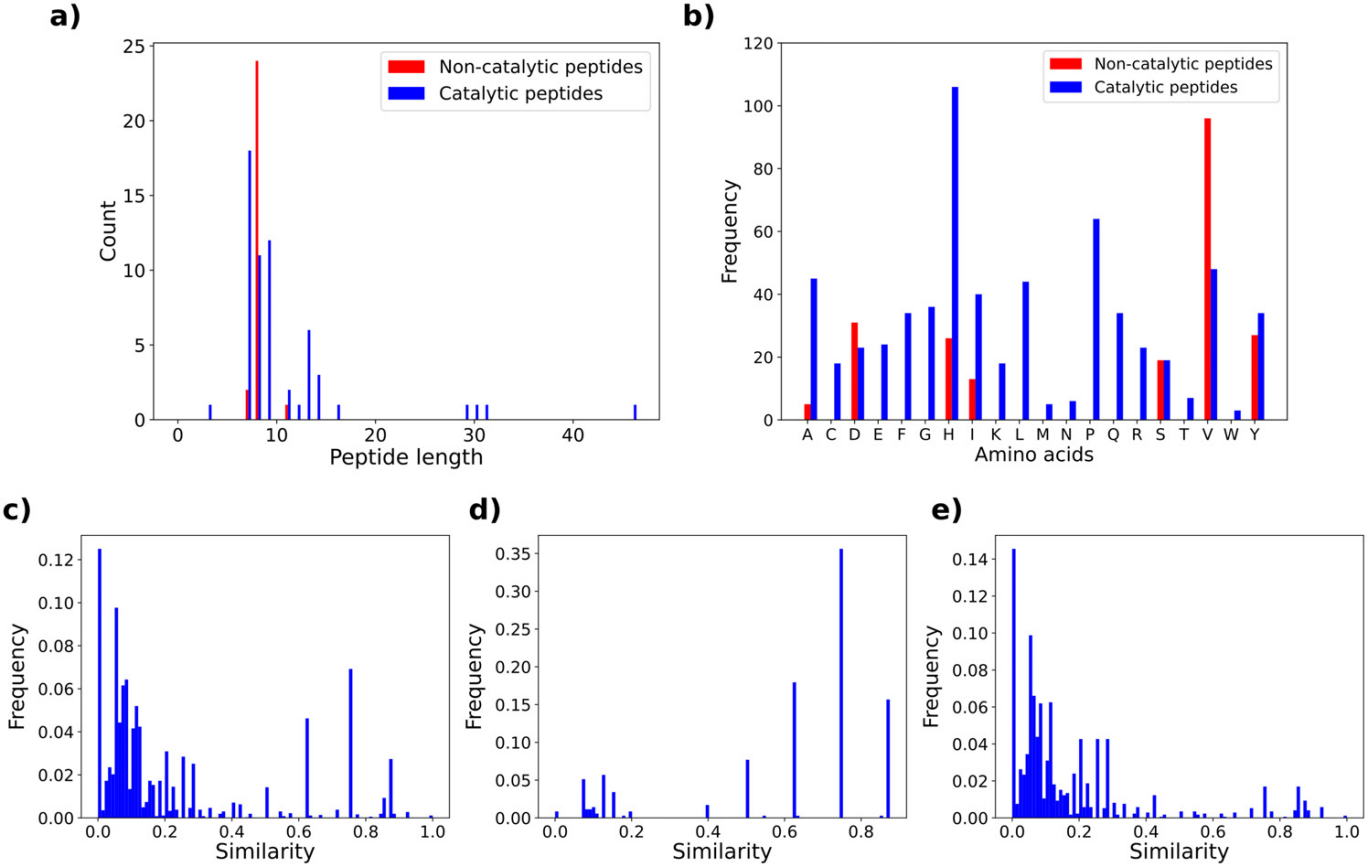
Statistical and physico-chemical properties computed for sequences active on p-NPA: (a) number of peptides with a specific combination of N- and C- termini and (b) distribution of peptides by catalysis mechanism. Distributions of examined physico-chemical properties: (c) charge on Lehninger scale at pH = 7.4, (d) hydrophobicity on Eisenberg scale and (e) isoelectric point on Lehninger scale.

**Fig. 2:**
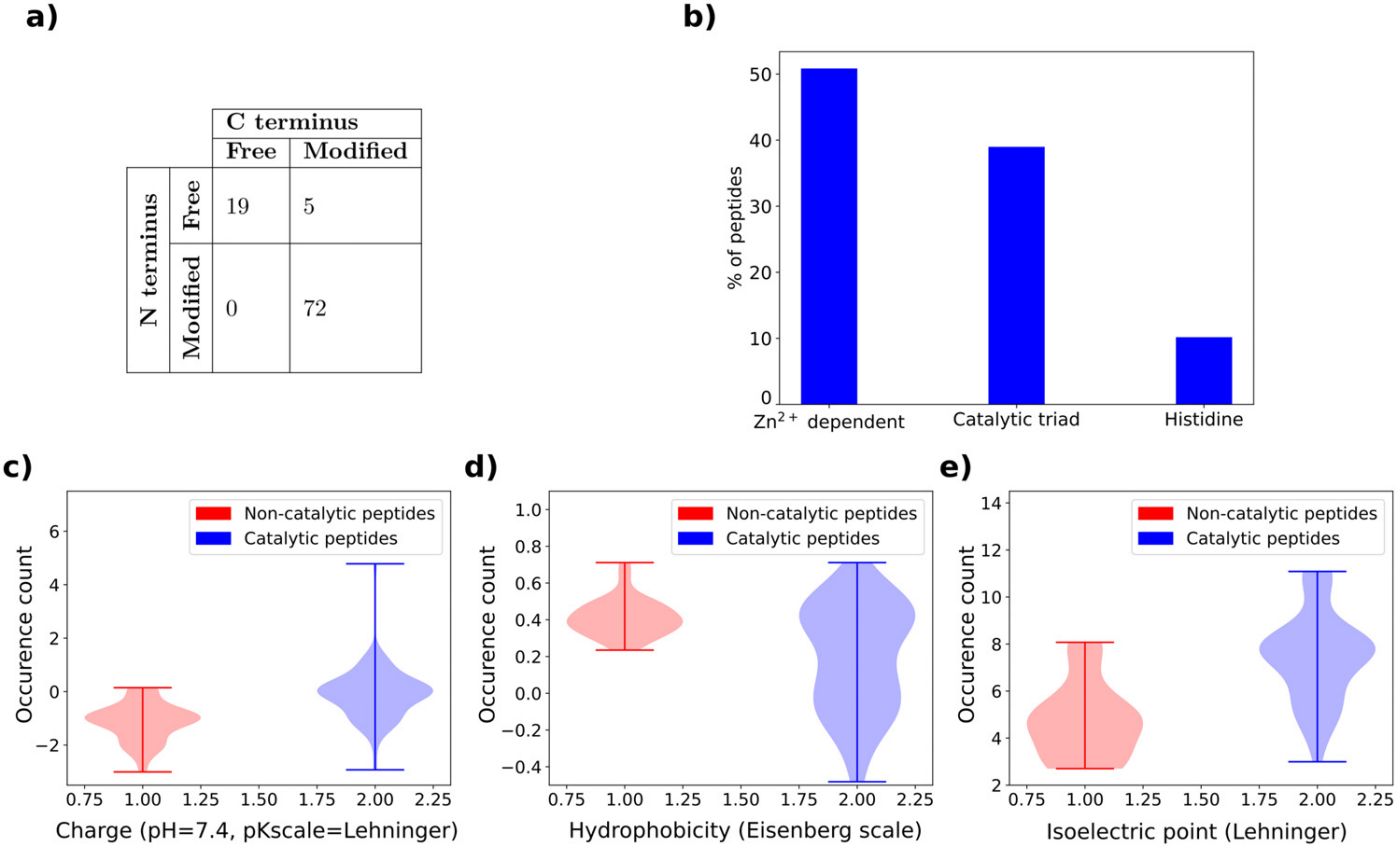
Statistical properties of sequences that catalyze the p-NPA substrate: (a) distribution of sequence lengths, (b) distribution of 20 proteinogenic amino acids. Similarity computed for pairs of peptides among: (c) all sequences, (d) inactive peptides and (e) active peptides.

Alongside the tables we performed a descriptive statistical analysis of peptides active on p-NPA, that represent the majority of entries. The violin plot representation was used to visualize the distribution of physico-chemical properties important for catalytic activity: charge at pH = 7.4 on the Lehninger scale (Fig. 1(c)), hydrophobicity on Eisenberg scale (Fig. 1(d)) and isoelectric point (Fig. 1(e)). Moreover, a table showing sequence termini modification combinations (Fig. 1(a)) and a histogram indicating the percentage of peptides depending on three main catalysis mechanisms (Fig. 1(b)) were presented for the catalytic peptides. Finally, the histogram of peptide lengths (Fig. 2(a)), the frequency analysis of proteinogenic amino acids reflecting the composition of peptides (Fig. 2(b)) and the similarity analysis of sequences (Fig. 2(c)–(e)) were provided for the active and inactive peptides.

## 3 Experimental Design, Materials and Methods

Data regarding catalytic peptides were collected from published articles up to the year 2023. Articles were searched in the academic search engine Google Scholar for the keywords catalytic peptides, p-NPA, p-NPP and self-assembly. The provided statistical analyses were performed only for peptides active on the p-NPA substrate with Python 3.8 programming language. All properties were separately analyzed for active and inactive sequences containing only proteinogenic amino acids. Distribution of amino acid residues (e.g. peptide length) was obtained by counting the number of peptides that are of specific length. Furthermore, we provide the overview of peptide compositions that was computed by counting how many times each amino acid occurs in the dataset. We also computed and analyzed the distributions of theoretical physico-chemical properties relevant to the catalytic activity with peptides.py 0.3.1 Python library. GRAVY hydrophobicity index of an amino acids sequence was computed using Eisenberg scale, by summing the hydrophobicity of individual amino acids and dividing this value by the length of the sequence. The net charge of the peptide sequence is computed by the Henderson–Hasselbalch equation at pH = 7.4 using Lehninger pKa scale. Isoelectric point, representing the pH level at which peptide carries no net charge, was computed for a Lehninger pKa scale. The peptide similarity was separately computed for the whole dataset, only for negative entries and only for positive entries. All pairs of peptides are aligned with the Needleman–Wunsch method from scikit-bio 0.5.8 Python library. The relative similarity representing the percentage of corresponding residues is computed for each pair of peptides in the dataset with respect to the longer peptide and thus achieves a value from a range [0, 1].

## Ethics Statement

Not applicable.

## CRediT authorship contribution statement

**Patrizia Janković:** Conceptualization, Methodology, Investigation, Writing – original draft. **Erik Otović:** Data curation, Visualization, Writing – original draft, Formal analysis. **Goran Mauša:** Visualization, Investigation, Supervision, Writing – review & editing. **Daniela Kalafatovic:** Conceptualization, Investigation, Supervision, Funding acquisition, Writing – review & editing.

## Declaration of Competing Interest

The authors declare that they have no known competing financial interests or personal relationships that could have appeared to influence the work reported in this paper.

## Data Availability

Catalytic Peptides Dataset (Reference data) (Mendeley Data).
